# MPC-Based Prediction of Anti-Mutant Effectiveness of Antibiotic Combinations: In Vitro Model Study with Daptomycin and Gentamicin against *Staphylococcus aureus*

**DOI:** 10.3390/antibiotics10101148

**Published:** 2021-09-23

**Authors:** Maria V. Golikova, Elena N. Strukova, Yury A. Portnoy, Stephen H. Zinner, Alexander A. Firsov

**Affiliations:** 1 Department of Pharmacokinetics & Pharmacodynamics, Gause Institute of New Antibiotics, 11 Bolshaya Pirogovskaya Street, 119021 Moscow, Russia; lena-stru@inbox.ru (E.N.S.); yaportnoy@gmail.com (Y.A.P.); kindyn@gmail.com (A.A.F.); 2 Department of Medicine, Mount Auburn Hospital, Harvard Medical School, 330 Mount Auburn St., Cambridge, MA 02138, USA; szinner@mah.harvard.edu

**Keywords:** daptomycin–gentamicin combination, in vitro model, anti-mutant effect

## Abstract

To explore whether combined treatments with daptomycin and gentamicin can prevent the development of *Staphylococcus aureus* resistance, and whether the possible restriction is associated with changes in antibiotic mutant prevention concentrations (MPCs), the enrichment of daptomycin- and gentamicin-resistant mutants was studied by simulating 5-day single and combined treatments in an in vitro dynamic model. The MPCs of the antibiotics in the combination were determined at concentration ratios equal to the ratios of 24 h areas, under the concentration–time curve (AUCs) of the antibiotics, as simulated in pharmacodynamic experiments. The MPCs of both daptomycin and gentamicin decreased in the presence of each other; this led to an increase in the time when antibiotic concentrations were above the MPC (*T*_>MPC_). The increases in *T*_>MPC_s were concurrent with increases of the anti-mutant effects of the combined antibiotics. When anti-mutant effects of the antibiotics in single and combined treatments were plotted against the *T*_>MPC_s, significant sigmoid relationships were obtained. These findings suggest that (1) daptomycin–gentamicin combinations prevent the development of *S. aureus* resistance to each antibiotic; (2) the anti-mutant effects of antibiotic combinations can be predicted using MPCs determined at pharmacokinetic-based antibiotic concentration ratios; (3) *T*_>MPC_ is a reliable predictor of the anti-mutant efficacy of antibiotic combinations.

## 1. Introduction

Emerging trends in antimicrobial resistance (AMR) presuppose the need to search for effective tools to improve treatment outcomes. One such tool is the use of combined therapy, with two or more antimicrobial agents, to mitigate the effects of AMR. Daptomycin is widely used to treat gram-positive infections including endocarditis and bacteremia, especially those caused by *Staphylococcus aureus* [1,2,3,4]. However, an increasing number of clinical case reports that document daptomycin resistance [5,6,7,8] highlight the necessity to explore combinations of daptomycin with other anti-staphylococcal antimicrobial agents, such as gentamicin. Currently, in vivo and in vitro data are limited with respect to the anti-mutant efficacy of this combination. The few extant in vivo studies have not shown that daptomycin–gentamicin combination is superior to daptomycin alone as applied to the suppression of resistance [9,10]; however, in one of the studied *S. aureus* strains, the development of daptomycin resistance was restricted in the presence of gentamicin [10].

Anti-mutant activity of the daptomycin–gentamicin combination was investigated in several in vitro studies, conducted in both static [11,12] and dynamic conditions [13,14,15]. The results of these studies yielded controversial conclusions, indicating that the anti-mutant efficacy of this combination might or might not be improved relative to daptomycin alone. In time-kill experiments, the daptomycin–gentamicin combination restricted the development of daptomycin resistance in *S. aureus* strains [11]. However, gentamicin could not prevent the appearance of daptomycin-resistant *S. aureus* cells when staphylococci were exposed to stepwise-increasing concentrations of daptomycin, alone or in combination with gentamicin [12]. In a pharmacodynamic study [13], the combination of daptomycin and gentamicin, at doses that correspond to therapeutic values, prevented the decrease in *S. aureus* susceptibility to both drugs noted in respective mono-treatments. However, in other studies [14,15], only the prevention of gentamicin resistance in the presence of daptomycin was detected, while resistance to daptomycin did not occur in mono- or combined treatments. These sparse data do not allow conclusions about daptomycin–gentamicin interactions in relation to anti-mutant effects. This supports the need for further investigation of the anti-mutant potential of daptomycin–gentamicin combinations.

A previous in vitro pharmacodynamic study of the combination of daptomycin with linezolid [16] revealed enhanced anti-mutant effects against *S. aureus*. The observed enhancement was attributed to lengthening the time within the dosing interval, when antibiotic concentrations exceeded the mutant prevention concentration (MPC) (*T*_>MPC_), as a result of lowering the MPCs of each antibacterial in the presence of the other. The MPCs of linezolid and daptomycin used in this combination were determined at concentration ratios equal to the ratio of the 24 h area under the concentration–time curve (AUC) of linezolid to the AUC of daptomycin simulated in the pharmacokinetic experiments. This allowed determination of the antibiotic MPCs used in the combination studies at pharmacokinetic-based concentration ratios.

To explore whether combined treatments with daptomycin and gentamicin can prevent the enrichment of *S. aureus* mutants resistant to both drugs, and if such restriction is associated with changes in antibiotic MPCs, the enrichment of daptomycin- and gentamicin-resistant mutants was studied by simulating 5-day single and combined treatments in an in vitro dynamic model. The exposures of daptomycin were simulated to provide subtherapeutic doses of the lipopeptide and to allow higher probability of daptomycin-resistant *S. aureus* mutant growth. In contrast, gentamicin-pharmacokinetics were simulated at therapeutic exposures, according to previous in vitro studies that reported high intensity of growth of gentamicin-resistant mutants, even at high-dose aminoglycoside regimens [13,15,17]. As referenced above [16], the MPCs of one antibiotic, in the presence of the other, were determined at a pharmacokinetic-based daptomycin-to-gentamicin concentration ratio, which was equal to the ratio of the AUC of daptomycin to the AUC of gentamicin in pharmacokinetic simulations of the combined treatments.

## 2. Results

### 2.1. MPCs of Daptomycin and Gentamicin Alone and in Combination

**Figure 1:**
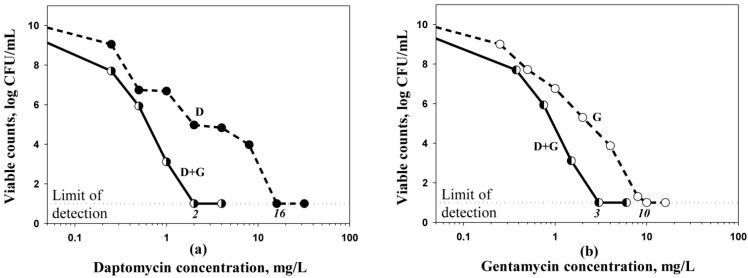
Determination of MPC of daptomycin (D) (**a**) and gentamicin (G) (**b**) alone and in combination at concentration ratio 1:1.5, respectively, against *S. aureus* 293.

MPCs were assessed for daptomycin and gentamicin alone and in combination (Table 1). Increasing antibiotic concentrations in the agar plates led to lower numbers of surviving cells. For each antibiotic, this lowering was more pronounced in the presence of the second agent. As an example, plots reflecting concentration-dependent changes in numbers of antibiotic-resistant mutants on agar plates with daptomycin or gentamicin, alone or in combination, at a daptomycin-to-gentamicin concentration ratio of 1:1.5, are shown in Figure 1. As seen in the figure, plots for daptomycin in the presence of gentamicin and vice versa are shifted to the left along the abscissa compared with those observed for each antibiotic alone. As a result, the estimated MPCs of daptomycin or gentamicin in combination were lower than the MPCs observed with the respective single agents. Under the influence of daptomycin, the MPCs of gentamicin decreased from 10 to 3–6 mg/L depending on the daptomycin-to-gentamicin concentration ratio (1.7–3.3-fold); under the influence of gentamicin, the MPCs of daptomycin decreased from 16 to 1.2-6mg/L depending on the daptomycin-to-gentamicin concentration ratio (2.7–13.3-fold).

### 2.2. Antibiotic Pharmacodynamics with Resistant S. aureus Mutants

**Figure 2:**
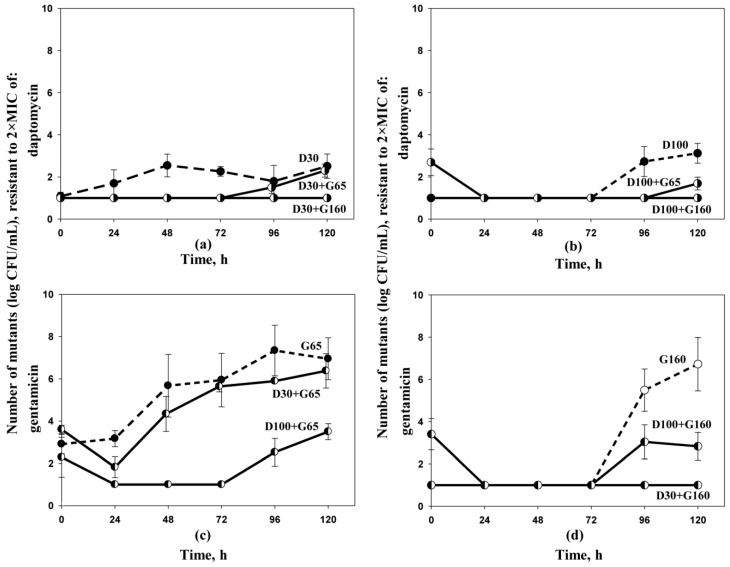
Time courses of subpopulations of *S. aureus* 293, resistant to 2 × MIC of daptomycin (**a**,**b**) and gentamicin (**c**,**d**). Dosing regimens are indicated at each curve. Data are presented as arithmetic means ± standard deviations.

Time courses of *S. aureus* mutants resistant to 2 × MIC of daptomycin and gentamicin are shown in Figure 2. As seen in the figure, at low exposure of daptomycin (regimen D30), *S. aureus* mutants resistant to 2 × MIC of antibiotic emerged after 24 h from the beginning of the experiment. At high daptomycin exposure (D100) the amplification of antibiotic-resistant cells was less pronounced, and occurred 72 h after beginning the experiment (Figure 2a,b). The growth of daptomycin-resistant cells was suppressed at regimens D30+G65 and D100+G65, and was completely restricted at regimens D30+G160 and D100+G160.

Gentamicin monotherapy at both AUCs, even at the super-therapeutic simulated dose, did not prevent the growth of mutants resistant to 2 × MIC of the antibiotic (Figure 2c,d). At gentamicin AUC of 65 mg × h/L the emergence of resistant *S. aureus* cells was pronounced and starting from 96 h reached 10^8^ CFU/mL, while in the presence of daptomycin the growth of gentamicin-resistant mutants was suppressed. The higher was the lipopeptide AUC in the combination, the lower were the numbers of gentamicin-resistant cells. However, even at regimen D100+G65, complete suppression of gentamicin-resistant cells was not observed. At gentamicin AUC of 160 proliferation of gentamicin-resistant mutants during monotherapy occurred starting from 72 h and reached 10^6^ CFU/mL at the end of the treatment. Similarly, an enhanced anti-mutant effect of the combination was observed at the higher gentamicin AUC (regimens D30+G160 and D100+G160). At regimen D30+G160, the growth of gentamicin-resistant mutants was completely restricted. Similar trends, showing enhanced anti-mutant efficacy of daptomycin–gentamicin combinations, were observed when bacterial growth was analyzed on agar plates with 4 × MIC of daptomycin and gentamicin (Figure S1, available as Supplementary data). The growth of resistant staphylococci on agar plates with 8 × MIC of daptomycin was not detected in either mono- or combined treatments, while gentamicin-resistant staphylococci were enriched in monotherapy with aminoglycoside and suppressed in the presence of daptomycin (data not shown).

**Figure 3:**
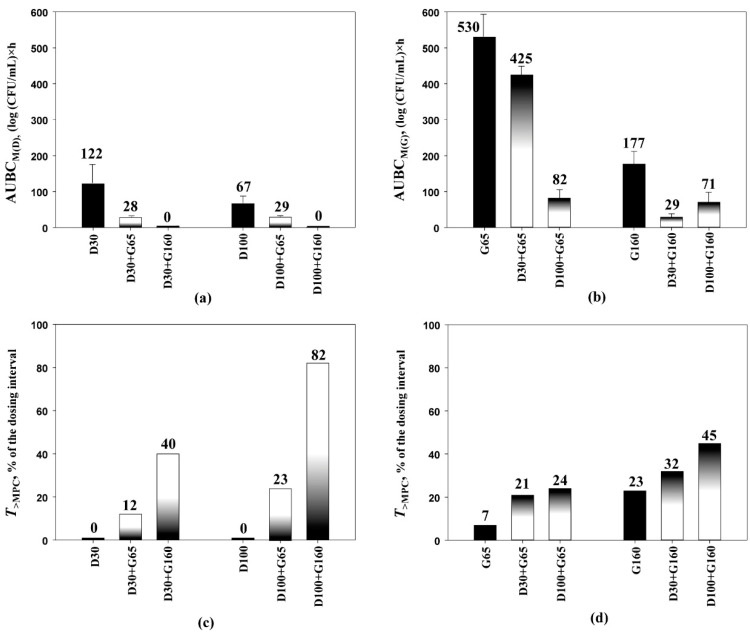
The enrichment of *S. aureus* 293 mutants resistant to 2 × MIC of daptomycin (**a**) or gentamicin (**b**) expressed as AUBC_M,_ and the respective values of *T*_>MPC_ (**c**,**d**). The parameter values are indicated over each bar. AUBC_M_ data are presented as arithmetic means ± standard deviations.

Enhancement of anti-mutant effects of daptomycin (expressed as the area under the bacterial mutant concentration–time curve, AUBC_M(D)_) in the presence of gentamicin was consistent with increases in *T*_>MPC_ levels (Figure 3a,c). For example, AUBC_M(D)_s declined from 122 (regimen D30) to 28 (log CFU/mL) × h (regimen D30+G65), along with the lengthening of *T*_>MPC_ from 0% to 12%, respectively. Changes in the anti-mutant effect of gentamicin (expressed as AUBC_M(G)_) under the influence of daptomycin are shown in Figure 3b,d. Again, the enhancement of gentamicin’s anti-mutant effect in the presence of daptomycin was accompanied by increased levels of *T*_>MPC_s. For example, when AUBC_M(G)_ decreased from 530 (regimen G65) to 425 (log CFU/mL) × h (regimen D30+G65), *T*_>MPC_s increased from 7% to 24%.

## 3. Discussion

In the current study, the anti-mutant potential of daptomycin–gentamicin combinations against *S. aureus* was examined. Daptomycin concentrations were simulated to achieve subtherapeutic AUCs, and to provide higher probability of the emergence of *S. aureus* resistance to daptomycin, while gentamicin was dosed to simulate therapeutic AUCs that correspond to once daily high-level doses of 5 and 7 mg/kg. Previous studies have documented that enrichment of gentamicin-resistant cells occurs even at high peak aminoglycoside concentrations [13,15,17]. As a result, when mono-treatments with daptomycin and gentamicin were simulated in the dynamic model, enrichment of resistant *S. aureus* mutants was observed. In contrast to monotherapy, all combined treatments revealed enhanced anti-mutant effectiveness against both daptomycin- and gentamicin-resistant *S. aureus*. This could be predicted from the increased values of *T*_>MPC_ associated with these regimens, which in turn were caused by lowered MPCs of both daptomycin and gentamicin in the presence of each other. The daptomycin-to-gentamicin concentration ratios, at which MPCs of antibiotics were determined, were equal to the respective daptomycin-to-gentamicin AUC ratios used in the pharmacokinetic simulations. This pharmacokinetic-based approach to MPC determination has been used in previous studies with the following antibiotic combinations: linezolid–rifampicin [18], daptomycin–rifampicin [19], linezolid–gentamicin [17] and linezolid–daptomycin [16]. In the current study the increased *T*_>MPC_s for each antibacterial in the presence of the second agent were consistent with enhanced anti-mutant effects of daptomycin and gentamicin in combination and consequently with decreased areas under the bacterial mutant concentration–time curve (AUBC_M_s) of daptomycin (AUBC_M(D)_) and gentamicin (AUBC_M(G)_), respectively. The present study demonstrates the protective effects of lengthening the times above MPC for daptomycin and gentamicin against the emergence of resistant *S. aureus* mutants. Similar conclusions, regarding the key role of increased *T*_>MPC_s of antibiotic combinations in enhancing their anti-mutant effects, were reported in our studies of combinations of daptomycin with linezolid [16] and linezolid with gentamicin [17] or rifampicin [18].

**Figure 4:**
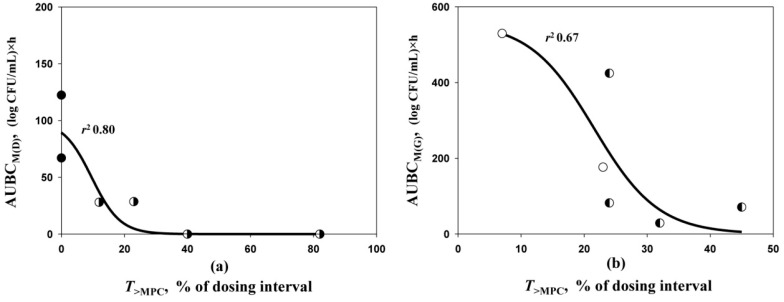
AUBC_M(D)_ (**a**) and AUBC_M(G)_ (**b**)—*T*_>MPC_ relationships fitted by Equation (1): Y_0_ = 0, x_0_ = 9.612, a = 100.4 and b = −4.515 (**a**); Y_0_ = 0, x_0_ = 21.56, a = 559.3 and b = −0.5106 (**b**). Filled symbols—daptomycin, empty symbols—gentamicin, semi-filled symbols—antibiotic combination.

To explore the relationships between daptomycin- and gentamicin-resistant *S. aureus* observed in mono- and combined treatments and their respective *T*_>MPC_s, AUBC_M_s were plotted against *T*_>MPC_s achieved in both single and combined treatments (merged data for each antibiotic) (Figure 4). Reasonable sigmoid *T*_>MPC_ relationships with AUBC_M(D)_ or AUBC_M(G)_ (*r*^2^ 0.80 or 0.67, respectively) were found. This indirectly suggests that MPCs determined at pharmacokinetic-based concentration ratios are predictive of the anti-mutant effectiveness of antibiotic combinations. The sigmoid shape of the relationships between resistance and *T*_>MPC_ has been reported previously with fluoroquinolone [20,21,22,23] and linezolid [24,25] monotherapy.

The anti-mutant efficacy of daptomycin–gentamicin combinations has been described previously in a study using an in vitro pharmacodynamic model with simulated endocardial vegetations [13]. Gentamicin addition at 5 mg/kg daily (the same as in current study) to daptomycin (6 mg/kg/day) prevented the emergence of resistance to both drugs relative to respective daptomycin and gentamicin monotherapy. In addition, in time-kill experiments, presented in another in vitro study, the daptomycin–gentamicin combination restricted the development of daptomycin resistance in *S. aureus* strains [11]. Our findings support these observations that daptomycin–gentamicin combinations can be effective in preventing staphylococcal resistance. Unfortunately, in a few other pharmacodynamic studies (therapeutic doses of daptomycin were simulated at 8 and/or 6 mg/kg/day) daptomycin resistance was not observed as the lipopeptide concentrations used (peak concentrations ranged from 80 to 133 mg/L) exhibited high bactericidal activity that did not allow exploration of possible improvement of the anti-mutant effect of daptomycin by gentamicin; at the same time, gentamicin resistance emerged in mono-treatments and was completely restricted in the presence of daptomycin [14,15].

It is worth noting that in the current study only one daptomycin-susceptible *S. aureus* strain was used. This limits the potential clinical relevance of our findings and suggests the need for further investigation of the anti-mutant potential of daptomycin–gentamicin combinations with a larger number of *S. aureus* strains. In addition, the clinical relevance of the current study is limited as we used subtherapeutic daptomycin doses. To improve the clinical significance of our research future simulation of therapeutic doses of daptomycin against *S. aureus* strains with decreased lipopeptide susceptibility should be considered. Another limitation is that the current study was carried out in in vitro conditions; this did not allow considerations of any effects of the immune system and/or protein binding, which might influence the anti-mutant efficacy of the antibiotics.

## 4. Materials and Methods

### 4.1. Antimicrobial Agents, Bacterial Strain and Susceptibility Testing

Daptomycin powder was purchased from Acros Organics (Fair Lawn, NJ, USA); gentamicin sulfate was purchased from PhytoTechnology Laboratories (Lenexa, KS, USA). Clinical isolate *S. aureus* 293 was used in the study; it was susceptible to both daptomycin and gentamicin with MICs of 0.5 and 0.25 mg/L, respectively.

### 4.2. MPC Determinations

Antibiotic MPCs, alone and in the presence of each other, were determined as described elsewhere [18]. Each experiment was conducted in triplicate. Daptomycin MPC in the presence of gentamicin and gentamicin MPC in the presence of daptomycin were determined at daptomycin-to-gentamicin concentration ratios equal to the respective antibiotic AUC ratios used in subsequent pharmacokinetic simulations. As a result, the daptomycin-to-gentamicin concentration ratios in MPC determinations were 1.5:1, 1:1.5, 1:2 and 1:5.

### 4.3. Antibiotic Dosing Regimens and Simulated Pharmacokinetic Profiles

Both single and combined treatments mimicked subtherapeutic dosing regimens of daptomycin, with respective AUCs of 30 mg × h/L (regimen D30, this designation was also used in combination treatments) and 100 mg × h/L (regimen D100), and therapeutic dosing regimens of gentamicin, 5 and 7 mg/kg once daily, with respective AUCs of 65 mg × h/L (regimen G65) and 160 mg × h/L (regimen G160) calculated using peak serum gentamicin concentrations reported in human studies (16.6 and 39.8 mg/L, respectively) [26,27]. Combined treatments with daptomycin and gentamicin were D100+G65 (1.5:1 ratio), D100+G160 (1:1.5 ratio), D30+G65 (1:2 ratio) and D30+G160 (1:5 ratio).

All pharmacodynamic experiments with once-daily dosing of daptomycin or gentamicin used alone or in combination were conducted for five consecutive days. The simulated antibiotic half-lives were as follows: 9 h for daptomycin [28] and 3 h [26] for gentamicin.

### 4.4. In Vitro Dynamic Model

To simulate mono-treatments with daptomycin and gentamicin a previously described dynamic model [29] was used. Briefly, the model consists of two connected flasks with fresh Mueller–Hinton broth supplemented with 50 mg of Ca^2+^/L (CSMHB), because daptomycin antimicrobial activity is influenced by the presence of Ca^2+^ [30]. One flask (central unit, volume 100 mL) also contained a magnetic stirrer and either a bacterial culture alone (growth control experiment) or a bacterial culture plus antibiotic (killing/regrowth experiments). Peristaltic pumps circulated fresh nutrient medium to and from the central unit, at a desired flow rate of 7.7 mL/h (for daptomycin) and 23.2 mL/h (for gentamicin) to provide a mono-exponential decay of antibiotic concentrations.

In combined treatments simultaneous mono-exponential elimination of daptomycin and gentamicin was simulated; the model was modified according to the Blaser and Zinner principle [31]. Briefly, the model was supplemented with an additional flask with fresh CSMHB (200 mL) and the antibiotic with the longer half-life, i.e., daptomycin, at a concentration equal to those in the central unit. Similar to mono-treatments, in combined treatments peristaltic pumps circulated fresh nutrient medium to and antibiotic-containing medium (with both daptomycin and gentamicin) from the central unit at a flow rate that corresponds to the antibiotic with the shorter half-life, i.e., gentamicin (23.2 mL/h). To compensate for a too rapid daptomycin loss from the central unit peristaltic pumps transferred the fresh medium with daptomycin from the additional flask to the central unit at a flow rate equal to the difference between the rates of gentamicin and daptomycin—15.5 mL/h (23.2 mL/h–7.7 mL/h).

The operation procedure used in the pharmacodynamic experiments was as described elsewhere [29]. Antibiotic dosing and sampling of the central unit were processed automatically using computer-assisted controls. Each experiment was performed at least in duplicate. Before the start of the experiment, the system was filled with sterile CSMHB, and placed in an incubator at 37 °C. After that, the central unit was inoculated with an 18-h culture of bacteria and incubated for several hours, until exponentially growing cultures reached ~10^8^ colony-forming units (CFU)/mL. Then antibiotics were administered into the central unit of the model. The duration of each experiment was 120 h.

### 4.5. Quantitation of the Antimicrobial Effects on the Resistant Subpopulations of S. aureus

To monitor the time courses of antibiotic-resistant subpopulations of *S. aureus* 293 in the pharmacodynamic experiments, the central unit of the model was multiply sampled throughout the observation period (120 h). The samples were serially diluted, if necessary, plated on Mueller–Hinton agar (MHA) with 2×, 4× and 8 × MIC of daptomycin or gentamicin, and incubated for up to 72 h at 37 °C. The viable counts were screened visually for growth. The lower limit of detection was 10 CFU/mL (equivalent to at least one colony per plate).

To characterize the time courses of *S. aureus* mutants resistant to 2 × MIC of antibiotic, the area under the bacterial mutant concentration–time curve (AUBC_M_) [32] was calculated from the onset to the end of the experiment (120 h) and corrected for the area under the lower limit of detection over the same time interval. For staphylococci resistant to daptomycin it was designed as AUBC_M(D);_ for staphylococci resistant to gentamicin it was designed as AUBC_M(G)_.

### 4.6. T_>MPC_ Relationships with the Emergence of Resistance

The AUBC_M(D)_s and AUBC_M(G)_s, determined in simulated mono- and combined treatments (merged data), were plotted against *T*_>MPC_ and fitted by the sigmoid function:

Y = Y0 + a/{1 + exp[−(x−x0)/b]},(1)

where Y is AUBC_M(D)_ or AUBC_M(G)_, x is *T*_>MPC_, Y_0_ and a are the minimal and maximal values of the AUBC_M_, respectively, x_0_ is x corresponding to a/2, and b is a parameter reflecting sigmoidicity.

All calculations were performed using SigmaPlot 12 software (Systat Software Inc., headquartered in San Jose, CA, USA).

## 5. Conclusions

The present study suggests that (1) daptomycin–gentamicin combination restricts the development of daptomycin- and gentamicin-resistant *S. aureus* mutants; (2) the inhibition of resistant *S. aureus* mutants using daptomycin–gentamicin combinations can be predicted by MPCs determined at pharmacokinetic-based antibiotic concentration ratios, and (3) *T*_>MPC_ is a reliable predictor of the anti-mutant efficacy of antibiotic combinations as studied using in vitro dynamic models.

## Figures and Tables

**Table 1:** MPCs (modal MPC estimation for a data set, *n* = 3) of daptomycin and gentamicin alone or in combination against *Staphylococcus aureus* 293. Measured MPC from each replicate was within one doubling of the modal MPC estimate.

Antibiotic	Daptomycin-to-Gentamicin AUC Ratio	Regimen	MPC, mg/L
Daptomycin	-	D30	16
		D100	16
Daptomycin in the presence of gentamicin	1:2	D30+G65	2
	1:5	D30+G160	1.2
	1.5:1	D100+G65	6
	1:1.5	D100+G160	2
Gentamicin	-	G65	10
		G160	10
Gentamicin in the presence of daptomycin	1:2	D30+G65	4
	1.5:1	D100+G65	4
	1:5	D30+G160	6
	1:1.5	D100+G160	3

## Data Availability

The majority of the data supporting the results of this study are located in the Supplementary Materials of this manuscript.

## Supplementary Materials

The following are available online at https://www.mdpi.com/article/10.3390/antibiotics10101148/s1, Figure S1: Time courses of subpopulations of *S. aureus* 293 resistant to 4×MIC of daptomycin (a,b) and gentamicin (c,d). Dosing regimens are indicated at each curve. Data are presented as arithmetic means ± standard deviations.
