# Supplementary material for: MPC-Based Prediction of Anti-Mutant Effectiveness of Antibiotic Combinations: In Vitro Model Study with Daptomycin and Gentamicin against Staphylococcus aureus
Source: Antibiotics (Basel). 2021 Sep 23;10(10):1148. doi: 10.3390/antibiotics10101148 (PMC8532831; doi:10.3390/antibiotics10101148)
Supplement: Supplementary file 1 [file antibiotics-10-01148-s001.zip › antibiotics-1363174-supplementary.pdf]

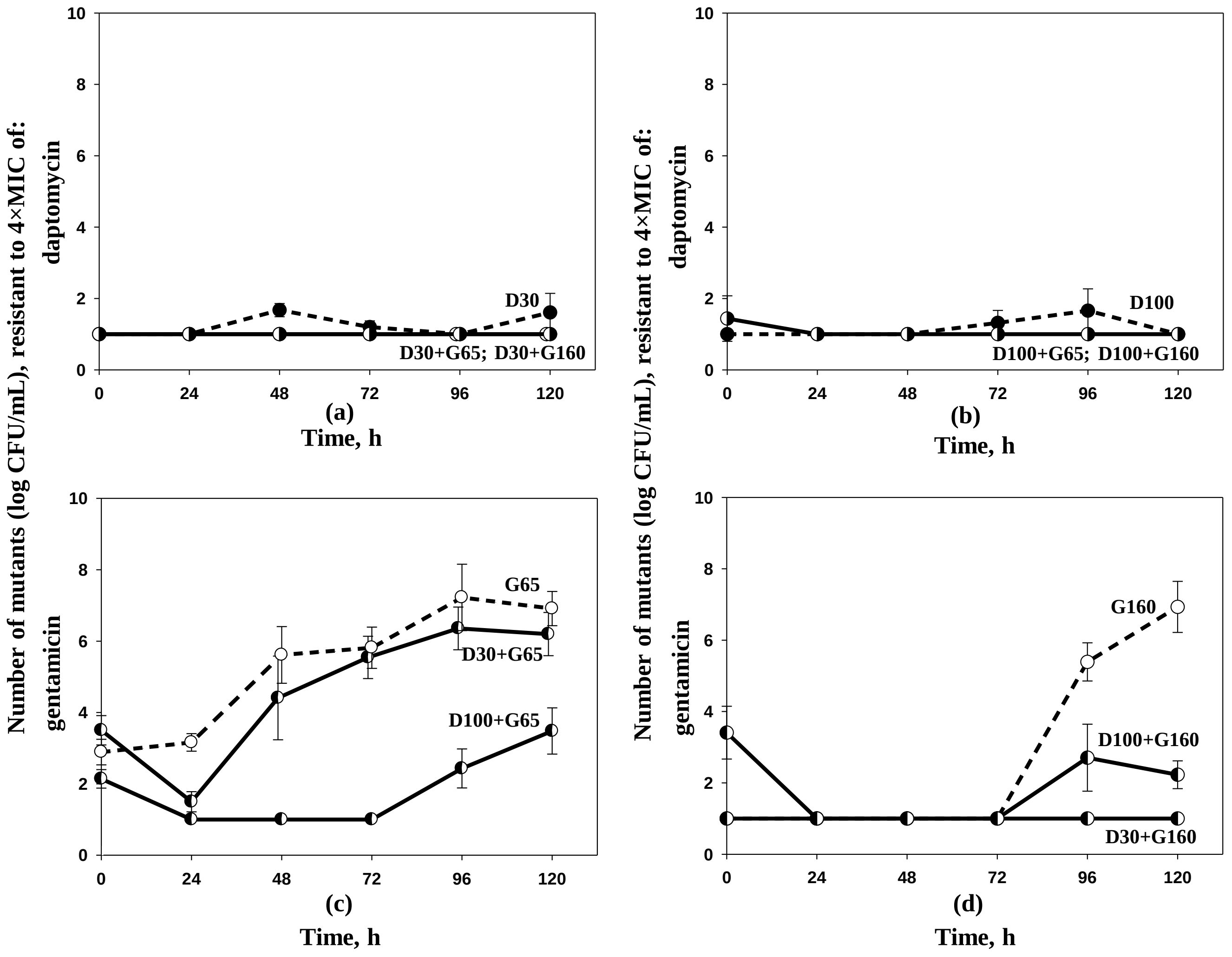

Fig. S1. Time courses of subpopulations of *S. aureus* 293 resistant to 4×MIC of daptomycin (a, b) and gentamicin (c, d). Dosing regimens are indicated at each curve. Data are presented as arithmetic means ± standard deviations.
